# The ability of pGCD59 to predict adverse pregnancy outcomes: a prospective study of non-diabetic pregnant women in Ireland

**DOI:** 10.1007/s00592-022-01983-z

**Published:** 2022-10-29

**Authors:** Delia Bogdanet, Michelle Toth Castillo, Helen Doheny, Louise Dervan, Miguel Angel Luque-Fernandez, Jose A. Halperin, Paula M. O’Shea, Fidelma P. Dunne

**Affiliations:** 1 College of Medicine, Nursing and Health Sciences, School of Medicine, National University of Ireland, Galway, Ireland; 2 Divisions of Haematology, Brigham & Women’s Hospital, Harvard Medical School, Boston, USA; 3 Department of Clinical Biochemistry, Saolta University Health Care Group (SUHCG), Galway University Hospitals, Galway, Ireland; 4 Department of Epidemiology, Harvard T.H. Chan School of Public Health, Boston, MA USA; 5 Department of Epidemiology and Population Health, London School of Hygiene and Tropical Medicine, London, UK

**Keywords:** Pregnancy outcomes, Gestational diabetes, Biomarker, pGCD59

## Abstract

**Aim:**

Even though most pregnancies are uneventful, occasionally complications do occur. Gestational diabetes is linked to an increased risk of adverse pregnancy outcomes. Early identification of women at risk of experiencing adverse outcomes, ideally through a single blood test, would facilitate early intervention. Plasma glycated CD59 (pGCD59) is an emerging biomarker which has shown promise in identifying hyperglycaemia during pregnancy and has been associated with the risk of delivering an LGA infant. The aim of this study was to explore the ability of the first- and second-trimester pGCD59 to predict adverse pregnancy outcomes.

**Methods:**

This was a prospective study of 378 pregnant women. Samples for pGCD59 were taken at the first antenatal visit and at the time of the 2 h 75 g OGTT (24–28 weeks of gestation). Adjusted receiver operating characteristic curves were used to evaluate the ability of pGCD59 to predict maternal and neonatal outcomes.

**Results:**

First-trimester pGCD59 levels were higher in women with gestational diabetes who delivered a macrosomic infant (4.2 ± 0.7 vs. 3.5 ± 1.0 SPU, *p* < 0.01) or an LGA infant (4.3 ± 0.3 vs. 3.6 ± 1.0 SPU, *p* = 0.01) compared to women with GDM that did not experience these outcomes. Second-trimester pGCD59 levels were higher in women that developed polyhydramnios (2.9 ± 0.4 vs. 2.5 ± 1.1 SPU, *p* = 0.03). First- and second-trimester pGCD59 predicted pregnancy-induced hypertension with good accuracy (AUC:0.85, 95%CI:0.78–0.91; AUC: 0.80, 95%CI: 0.73–0.88, respectively) and neonatal hypoglycaemia with fair to good accuracy (AUC:0.77, 95%CI: 0.54–0.99, AUC:0.81, 95%CI:0.62–0.99).

**Conclusions:**

This study has shown that pGCD59 has the potential to predict adverse pregnancy outcomes. Prospective studies with a larger number of cases are necessary to fully explore and validate the potential of this emerging biomarker in predicting adverse pregnancy outcomes.

## Introduction

Pregnancy is an important but delicate period for both mother and infant. Although the majority of pregnancies are uneventful, complications do arise affecting both the mother and the child. Pregnancies affected by gestational diabetes (GDM), however, are associated with an increased risk of developing adverse pregnancy outcomes. GDM has been correlated with an increased risk of developing hypertensive disorders of pregnancy (HDP) [1, 2] and a twofold increased risk in delivery by caesarean section [3, 4]. Polyhydramnios and oligohydramnios have also been linked with pregnancies complicated by diabetes [5, 6]. GDM has also been associated with a twofold to fourfold increased risk in having post-partum haemorrhage in the index pregnancy [7, 8] and with a twofold increased risk of antepartum haemorrhage [9, 10]. This might be secondary to the development of HDP which may lead to vascular ruptures or placental abruption.

The effect of maternal hyperglycaemia on the foetus can be explained by the Pendersen hypothesis [11]. Elevated maternal glucose levels lead to an increased transplacental transfer of glucose to the infant. This, in turn, stimulates foetal pancreatic *β*-cells to secrete insulin which leads to foetal macrosomia. Numerous studies support the association between GDM and macrosomia/large for gestational age (LGA) [12–14]. Shoulder dystocia has also been linked to GDMs. As infants of women with diabetes are more likely to be macrosomic, this increases the risk of shoulder dystocia or birth injury during normal delivery [15]. Vasculopathy due to hyperglycaemia and increased insulin secretion can lead to intrauterine growth restriction (IUGR) and small for gestational age (SGA) [16]. Hedderson et al*.* [17] found that maternal hyperglycaemia was associated with an increased risk of prematurity. The higher rate of adverse outcomes in infants of women with GDM inevitably leads to an increase in rates of neonatal intensive care (NICU) admissions in this cohort [18].

Antepartum identification of women at risk of developing a particular outcome, ideally through a single blood test, would facilitate early treatment, close monitoring, and the peri-partum implementations of adequate systems to ensure a safe delivery. The ability to predict adverse pregnancy outcomes may also help gain knowledge into the pathophysiological mechanisms involved in the development of pregnancy complications. Therefore, there is increased interest in the research of maternal biomarkers.

Plasma glycated CD59 (pGCD59) is an emerging biomarker which has shown promise in identifying hyperglycaemia during pregnancy [19]. Given its potential, researchers have also explored its ability to predict adverse pregnancy outcomes, particularly GDM-related adverse pregnancy outcomes, but research to date is limited. Ghosh et al*.* [19] found that higher pGCD59 levels were associated with higher LGA prevalence independent of maternal age and body mass index (BMI). Ma et al. [20] also found that higher maternal levels of pGCD59 were associated with the risk of delivering an LGA baby. Meek et al. [21], in a type 1 diabetes (T1DM) cohort, found that pGCD59 was associated with preterm birth, LGA and NICU admissions.

In this study, we aimed to explore the ability of the first (T1)- and second (T2)-trimester pGCD59 to predict adverse pregnancy outcomes in a pregnant cohort that included both normoglycaemic women and women diagnosed with GDM at 24–28 weeks of gestation (WG) with a 2 h 75 g oral glucose tolerance test (OGTT) and diagnosed by the 2013 World Health Organization (WHO) criteria.

## Methods

The protocol for this study has been published [22]. Between November 2018 and March 2020, this prospective study recruited pregnant women who had their first antenatal visit at Galway University Hospital in Galway, Ireland. Only pregnant women without pre-established diabetes were invited to take part in the study. At the first antenatal consultation, the patient information leaflet (PIL) was distributed, and a member of the research team described the study's goal and procedures. If agreeable, a consent form was signed.

At the first antenatal visit, the women's weight and height were measured on SECA scales model 799 (22,089 Hamburg, Germany), and the BMI was calculated. A mobile blood pressure monitor was used to measure maternal blood pressure (SECA mVSA 535). An ultrasound was used to confirm the gestational age of the women.

Women were screened for GDM in the second trimester (24–28 WG). One abnormal plasma glucose value in the OGTT according to WHO standards (fasting value 5.1 mmol/L (92 mg/dl), 1-h value 10 mmol/L (180 mg/dl), and 2-h value 8.5 mmol/L (153 mg/dl)) was used to define GDMs [23]. For plasma glucose measurement, whole blood was taken in fluoride oxalate specimen tubes, and glucose was tested using the hexokinase technique on the Roche Cobas® 8000 analyser (Roche Diagnostics, Indianapolis, USA).

Samples for pGCD59 measurements were taken at the first antenatal visit together with routine bloods and again at the time of the routine 2 h 75 g OGTT. At each sampling, blood (10 mL) was collected into ethylenediaminetetraacetic acid (EDTA). Each pGCD59 plasma sample was split into two 500L aliquots with barcodes and stored at −80 ℃. To ensure participant confidentiality, all laboratory specimens were assigned a coded identity number. The barcoded samples were linked to a clinical database that was pseudo-anonymized. This information was password-protected and stored on a secure server. Once the recruitment stage was completed, an aliquot of each participant's EDTA plasma sample was transferred on dry ice to the Laboratory for Translational Research, Haematology Division, Department of Medicine, Brigham and Women's Hospital, Boston, USA, for pGCD59 analysis. The enzyme-linked immunosorbent assay (ELISA) test previously described by Gosh et al*.* [24] was used to detect pGCD59. The coefficient of variability (CV) of the intraassay was 3.0%. The women's glucose tolerance was undisclosed to the test operators.

### Variables definition

LGA is defined as an infant birth weight according to gestational age and sex greater than or equal to the 90th percentile on a standard growth chart and macrosomia as an infant birth weight greater than or equal to 4000g [25]. SGA is defined as an infant birth weight less than or equal to the 10th percentile for gestational age and sex on a standard growth chart [26]. LGA and SGA were calculated using the Global Bulk Centile Calculator (BCC version 8.0.6.1) developed by the Perinatal Institute, Birmingham, UK. Prematurity was defined as a baby born alive before 37 completed weeks of pregnancy [27]. Mortality included stillbirth and early neonatal death (first 7 days). Preeclampsia was defined as new onset systolic blood pressure (SBP) of at least 140 mmHg and/or diastolic blood pressure (DBP) of at least 90 mmHg at more than 20 weeks’ gestation with proteinuria of greater than 300 mg/day [28]. Pregnancy-induced hypertension (PIH) was defined as new-onset blood pressure (BP) at least 140/90mmHg after 20 weeks gestation with no proteinuria. HDP included both preeclampsia and PIH. The decision to proceed with a caesarean delivery was made by the woman’s obstetrician. Polyhydramnios was diagnosed when the amniotic fluid index measured is greater than 24cm on foetal ultrasound on one or more occasion [29, 30]. Oligohydramnios refers to amniotic fluid volume that is less than the minimum expected for gestational age [31]. Shoulder dystocia was described as foetal shoulders not delivering after the head has emerged from the mother’s introitus due to either one or both shoulders becoming impacted against the bones of the maternal pelvis [32]. Neonatal hypoglycaemia (NH) was defined as a plasma venous glucose level of less than 2.6 mmol/L [33, 34] in the first 24h postpartum. In our centre, infants are tested for glucose levels only if they are symptomatic or in NICU.

Maternal composite outcomes included postpartum haemorrhage (PPH), antepartum haemorrhage (APH), HDP, polyhydramnios, and oligohydramnios.

Neonatal composite outcomes included LGA, macrosomia, SGA, prematurity, NICU admission, mortality, jaundice, and shoulder dystocia.

### Statistical analysis

We used mean and standard deviations/median and interquartile range to describe the continuous variables, while for categorical variables, we used count and percentages. We described missing data and explored the missing data mechanisms (i.e., missing completely at random, missing at random, and missing not at random). We used the *χ*^2^ test for categorical variables, the Wilcoxon–Mann–Whitney test for continuous variables not normally distributed, and Student’s t tests for continuous variables normally distributed to compare baseline characteristics of pregnant women with normal glucose tolerance (NGT) to baseline characteristics of pregnant women who developed GDM. Delta pGCD59 (*Δ*pGCD59) was calculated as the difference in pGCD59 levels between the first and second trimester of pregnancy.

Adjusted receiver operating characteristic (ROC) curves for GDM status, maternal age, BMI, maternal ethnicity, parity, previous GDM, and family history of diabetes were used to evaluate the ability of pGCD59 to predict maternal and neonatal outcomes. Then, the respective area under the curve (AUC) and 95% confidence interval (CI) were calculated. Diagnostic accuracy measures (sensitivity, specificity, positive predictive value (PPV), and negative predictive value (NPV)) were estimated and presented with 95%CIs.

The power calculation and sample size have been previously described [22]. In all analyses, a *P*-value < 0.05 was considered statistically significant. SPSS for Windows, version 20, was used for all statistical analyses (IBM SPSS Statistics for Windows, version 20 SPSS, Chicago, IL).

## Ethics

Ethical approval for this study was granted by the Clinical Research Ethics Committee, Galway, Ireland (Reference No- C.A. 2026).

## Results

**Fig. 1:**
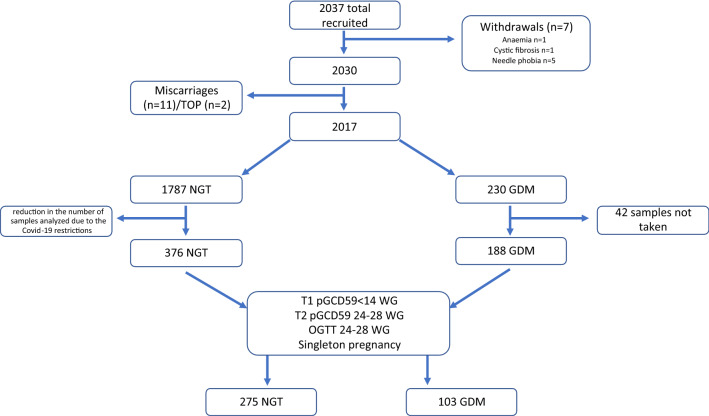
Study flow chart

Of the 2,037 subjects enrolled in this study, 7 withdrew, 11 had miscarriages, and 2 underwent pregnancy termination (TOP). Reasons for withdrawal included anaemia (*n* = 1), cystic fibrosis (*n* = 1), and needle fear (*n* = 5) (Fig. 1). GDM was identified in 230 of the remaining 2017 participants, 42 of whom did not have samples taken at both the first antenatal visit and at the time of the OGTT. The remaining 188 women with GDM together with 376 pregnant women with NGT matched for age, BMI and ethnicity and having samples collected at the first antenatal visit and again at the time of the OGTT provided for a cohort of 564 participants. From these 564 women, only those women meeting the following criteria: first sample (T1) taken at < 14 WG, a second sample (T2), an OGTT at weeks 24–28 WG, and singleton pregnancy (NGT *n* = 275, GDM *n* = 103) were selected. Missing data were assumed to be completely at random, and we performed a complete case analysis. Table 1 shows the participants' characteristics as well as the results of their laboratory tests. When compared to women with NGT, women with GDM had higher SBP (p = 0.03) and mean BP (*p* = 0.02). There were no other differences in the baseline characteristics of the two groups. Women with GDM had significantly higher T2 pGCD59 levels (*p* = 0.003). In comparison with the GDM cohort, ΔpGCD59 was greater in women with NGT (*p* = 0.01). As expected, women with GDM had higher glucose levels at all time points on the OGTT).

**Table 1:** Women’s baseline characteristics and laboratory values at Galway University Hospital, Galway, Ireland, between November 2018 and March 2020, n = 378

	NGT *n* = 275 (IQR/%)	GDM *n* = 103(IQR/%)	*P* value
*Baseline characteristics*			
Age (years)	33.6 (31.1–36.4)	34.8 (31.7–37.4)	0.07
WG at booking	12.7 (12–13.1)	12.4(12–13.1)	0.62
Gravida	2 (1–3)	2(1–3)	0.31
Parity	1 (0–1)	1(0–2)	0.63
Height (cm)	165 (161.6–169.5)	164(160–169)	0.12
Weight (kg)	73 (64–86.2)	75.7(64.2–89.6)	0.34
BMI (kg/m^2^)	26.4 (23.3–31)	28.7(23.7–31.9)	0.14
Ethnicity (white)	243/275 (88.4)	88/103 (85.4)	0.62
SBP (mmHg)	120 (112–126)	122(114–130)	**0.03**
DBP (mmHg)	69 (62–75)	69(64–79)	0.11
Mean BP (mmHg)	86 (80.6–91)	88.3(79.6–94.3)	**0.02**
WG at delivery	40 (39–40.8)	39.4(38.8–40.4)	0.07
Alcohol at booking	4/275 (1.4)	1/103 (0.9)	0.21
Alcohol before pregnancy	233/275 (84.7)	81/103 (78.6)	0.33
Non-smoker	145/275 (52.7)	55/103 (53.3)	0.92
Smoker at booking visit	14/275 (5)	8/103 (7.7)	0.21
*Laboratory values*			
T1pGCD59 (SPU)	3.6 (2.8–4.4)	3.7 (2.9–4.5)	0.92
T2pGCD59 (SPU)	2.39 (1.85–2.9)	2.6 (1.9–3.4)	** < 0.01**
ΔpGCD59	1.2 (0.4–2)	1.1 (0.09–1.7)	**0.01**
OGTT 24–28 weeks:			
Fasting glucose (mmol/L)	4.4 (4.2–4.6)	5.1(4.6–5.3)	** < 0.01**
1-h glucose (mmol/L)	7 (5.8–7.9)	10 (8.7–10.8)	** < 0.01**
2-h glucose (mmol/L)	5.5 (4.8–6.4)	7.1 (6–8.7)	** < 0.01**
Mean glucose (mmol/L)	5.5 (5.1–6.1)	7.2 (6–8.7)	** < 0.01**

*BMI*: Body mass index; *BP*: blood pressure; *DBP*: diastolic blood pressure; *GDM*: gestational diabetes; *IQR*: interquartile range; *NGT*: normal glucose tolerance; *OGTT*: oral glucose tolerance test; *SBP*: systolic blood pressure; T1: first trimester; *WG*: weeks of gestation. Missing data: SBP/DBP: *n* = 4

Bold values denote statistical significance at the *P* < 0.5 level

Pregnancy outcomes by glucose status are presented in Table 2. Women with GDM had higher rates of oligohydramnios (1.9% vs. 0%, *p* = 0.02) compared to women with NGT. There were no other statistically significant differences between the women with GDM and those with NGT for adverse maternal outcomes. Infants of women with GDM were more likely to develop post-partum jaundice (4% vs 0.7%) and to suffer shoulder dystocia at birth (2% vs. 0%) compared to infants of women with NGT. There were no other statistically significant differences between the infants of women with GDM and those of women with NGT for adverse infant outcomes.

**Table 2:** Pregnancy outcomes at Galway University Hospital, Galway, Ireland, between November 2018 and March 2020, *n* = 378

Outcome	NGT *n* = 275 (IQR/%)	GDM *n* = 103(IQR/%)	*P* value
*Mother*			
Total blood loss (mL)	350 (300–500)	350 (300–500)	0.11
PPH	71/270 (26.2%)	31/98 (31.6%)	0.32
Major PPH	5/270 (1.8%)	4/98 (4%)	0.22
Emergency CS	48/272 (17.6%)	15/98 (15.3%)	0.67
Elective CS	50/272 (18.3%)	26/98 (26.5%)	0.07
Preeclampsia	1/275 (0.36%)	2/103 (1.9%)	0.11
PIH	17/275 (6.1%)	4/103 (3.8%)	0.36
HDP	18/275 (6.5%)	6/102 (5.8%)	0.75
APH	4/275 (1.4%)	2/103 (1.9%)	0.71
Polyhydramnios	10/275 (3.6%)	2/103 (1.9%)	0.44
Oligohydramnios	0/275 (0%)	2/103 (1.9%)	**0.02**
Composite maternal	67/272 (24.6%)	23/99 (23.2%)	0.82
*Infant*			
Prematurity	9/272 (3.3%)	3/99 (3%)	0.81
Baby gender (female)	134/272 (49.2%)	45/99 (45.4%)	0.55
Baby weight	3600 (3265–3900)	3540 (3240–3880)	0.48
Macrosomia	45/272 (16.5%)	17/99 (17.1%)	0.81
Major macrosomia	6/272 (2.2%)	2/99 (2%)	0.98
LGA	28/272 (10.3%)	5/99 (5%)	0.11
SGA	32/272 (11.7%)	9/99 (9%)	0.42
Baby length	52 (50–54.5)	52 (50–54)	0.23
Head circumference	35 (34–36)	35 (33.3–35.5)	0.81
NICU admission	33/272 (12.1%)	10/99 (10.1%)	0.52
Jaundice	2/272 (0.7%)	4/99 (4%)	**0.02**
Neonatal death	2/272 (0.7%)	0/99 (0%)	0.44
Shoulder dystocia	0/272 (0%)	2/99 (2%)	**0.01**
Anomaly at birth	1/272 (0.36%)	0/98 (0%)	0.54
Apgar score at 1 min	9 (9–9)	9 (9–9)	0.32
Apgar score at 5 min	9 (9–9)	9 (9–9)	0.17
Composite neonatal	104/272 (38.2%)	34/99 (34.3%)	0.54

*APH*: Antepartum haemorrhage; *CS*: caesarean section; *GDM*: gestational diabetes; *IQR*: interquartile range; *HDP*: hypertensive disorders of pregnancy; *LGA*: large for gestational age; *NGT*: normal glucose tolerance; *NICU*: neonatal intensive care unit; *PIH*: pregnancy-induced hypertension; *PPH*: postpartum haemorrhage; *SGA*: small for gestational age

Bold values denote statistical significance at the *P* < 0.5 level

We assessed pGCD59 levels distribution across pregnancy outcomes in the entire cohort, in women with NGT alone and in women with GDM alone (Table 3).

**Table 3:** pGCD59 level distribution across pregnancy outcomes at Galway University Hospital, Galway, Ireland, between November 2018 and March 2020, *n* = 378

Outcome	Total cohort			NGT			GDM		
	Yes	No	*p*-value	Yes	No	*p*-value	Yes	No	*p*-value
T1 mean pGCD59 ± SD									
*Mother*									
PPH	3.6 ± 1.1	3.6 ± 1.1	0.71	3.5 ± 1.2	3.7 ± 1.1	0.31	3.8 ± 0.9	3.6 ± 1.0	0.22
Preeclampsia	4.0 ± 1.3	3.6 ± 1.1	0.60	2.8 ± 0.1	3.6 ± 1.1	0.42	4.6 ± 1.3	3.6 ± 1.0	0.20
PIH	3.6 ± 1.1	3.6 ± 1.1	0.88	3.6 ± 1.2	3.6 ± 1.1	0.92	3.5 ± 1.0	3.6 ± 1.0	0.71
HDP	3.7 ± 1.1	3.6 ± 1.1	0.97	3.6 ± 1.2	3.6 ± 1.1	0.88	3.8 ± 1.1	3.6 ± 1.0	0.61
APH	3.0 ± 0.7	3.7 ± 1.1	0.23	3.0 ± 0.9	3.7 ± 1.1	0.32	3.1 ± 0.8	3.6 ± 1.0	0.40
Polyhydramnios	3.7 ± 1.5	3.7 ± 1.1	0.71	3.7 ± 1.7	3.6 ± 1.1	0.91	4.1 ± 0.1	3.6 ± 1.0	0.51
Oligohydramnios	3.9 ± 0.4	3.6 ± 1.1	0.70	N/A	N/A	N/A	3.9 ± 0.4	3.6 ± 1.0	0.73
Composite maternal	3.5 ± 1.1	3.7 ± 1.1	0.22	3.5 ± 1.1	3.7 ± 1.1	0.31	3.5 ± 1.0	3.7 ± 1.0	0.41
*Infant*									
Prematurity	3.6 ± 0.7	3.7 ± 1.1	0.71	3.4 ± 0.8	3.7 ± 1.1	0.42	4.0 ± 0.4	3.6 ± 1.0	0.50
Macrosomia	3.5 ± 0.9	3.7 ± 1.1	0.33	3.3 ± 0.9	3.7 ± 1.1	**0.01**	4.2 ± 0.7	3.5 ± 1.0	** < 0.01**
LGA	3.4 ± 1.1	3.7 ± 1.1	0.11	3.2 ± 1.1	3.7 ± 1.1	**0.04**	4.3 ± 0.3	3.6 ± 1.0	**0.01**
SGA	3.8 ± 1.3	3.6 ± 1.0	0.40	3.7 ± 1.4	3.6 ± 1.1	0.90	4.2 ± 0.9	3.6 ± 1.0	0.13
NICU admission	3.5 ± 1.2	3.7 ± 1.0	0.55	3.5 ± 1.4	3.7 ± 1.1	0.45	3.6 ± 0.8	3.6 ± 1.0	0.91
Jaundice	3.9 ± 0.4	3.6 ± 1.1	0.62	3.8 ± 0.5	3.6 ± 1.1	0.86	3.9 ± 0.4	3.6 ± 1.0	0.65
Neonatal death	3.0 ± 0.3	3.6 ± 1.1	0.42	3.0 ± 0.3	3.6 ± 1.1	0.47	N/A	N/A	N/A
Shoulder dystocia	4.7 ± 0.03	3.6 ± 1.1	** < 0.01**	N/A	N/A	N/A	4.7 ± 0.1	3.6 ± 1.0	< **0.01**
Composite neonatal	3.6 ± 1.1	3.6 ± 1.0	0.98	3.5 ± 1.2	3.7 ± 1.1	0.07	4.1 ± 0.8	3.4 ± 1.0	** < 0.01**
T2 mean pGCD59 ± SD									
*Mother*									
PPH	2.5 ± 1.0	2.5 ± 1.2	0.72	2.4 ± 1.0	2.4 ± 0.9	0.60	2.8 ± 1.5	2.9 ± 1.2	0.70
Preeclampsia	2.7 ± 1.4	2.5 ± 1.1	0.80	1.1 ± 0.01	2.4 ± 1	0.16	3.5 ± 0.6	2.8v1.3	0.34
PIH	2.1 ± 0.7	2.6 ± 1.1	**0.03**	2.1 ± 0.8	2.4 ± 1	0.28	2.2 ± 0.4	2.8 ± 1.3	0.05
HDP	2.2 ± 0.8	2.6 ± 1.1	0.07	2.0 ± 0.8	2.4 ± 1.0	0.17	2.6 ± 0.7	2.8 ± 1.3	0.51
APH	1.7 ± 1.0	2.5 ± 1.1	0.07	2.1 ± 1.0	2.4 ± 1.0	0.57	0.9 ± 0.02	2.9 ± 1.3	** < 0.01**
Polyhydramnios	2.9 ± 0.4	2.5 ± 1.1	**0.03**	2.8 ± 0.4	2.4 ± 1.0	**0.04**	3.5 ± 0.1	2.8 ± 1.3	** < 0.01**
Oligohydramnios	1.7 ± 0.03	2.5 ± 1.1	** < 0.01**	N/A	N/A	N/A	1.7 ± 0.03	2.8 ± 1.3	** < 0.01**
Composite maternal	2.5 ± 1.1	2.5 ± 1.1	0.63	2.5 ± 0.9	2.4 ± 1.0	0.61	2.5 ± 1.4	2.9 ± 1.2	0.11
*Infant*									
Prematurity	2.4 ± 0.7	2.5 ± 1.1	0.61	2.4 ± 0.8	2.4 ± 1.0	0.83	2.5 ± 0.2	2.8 ± 1.3	0.07
Macrosomia	2.6 ± 1.1	2.5 ± 1.1	0.51	2.5 ± 1.1	2.4 ± 0.9	0.33	2.8 ± 1.2	2.8 ± 1.3	0.90
LGA	2.3 ± 0.8	2.6 ± 1.1	0.18	2.2 ± 0.7	2.5 ± 1.0	0.17	2.9 ± 1.4	2.8 ± 1.3	0.85
SGA	2.4 ± 0.8	2.6 ± 1.1	0.38	2.3 ± 0.8	2.4 ± 1.0	0.37	2.7 ± 0.8	2.8 ± 1.3	0.82
NICU admission	2.3 ± 0.8	2.6 ± 1.1	**0.04**	2.3 ± 0.8	2.4 ± 1.0	0.44	2.2 ± 0.7	2.9 ± 1.3	**0.03**
Jaundice	2.5 ± 0.2	2.5 ± 1.1	0.91	2.5 ± 0.4	2.4 ± 1.0	0.91	2.5 ± 0.1	2.8 ± 1.3	**0.04**
Neonatal death	2.0 ± 1.2	2.5 ± 1.1	0.44	2.0 ± 1.2	2.4 ± 1.0	0.50	N/A	N/A	N/A
Shoulder dystocia	2.7 ± 0.08	2.5 ± 1.1	0.81	N/A	N/A	N/A	2.6 ± 0.8	2.8 ± 1.3	0.23
Composite neonatal	2.5 ± 0.9	2.6 ± 1.1	0.42	2.4 ± 0.9	2.4 ± 1.0	0.71	2.7 ± 1.0	2.9 ± 1.4	0.53
ΔpGCD59									
*Mother*									
PPH	1.1 ± 1.4	1.1 ± 1.4	0.96	1.1 ± 1.3	1.2 ± 1.3	0.51	1.0 ± 1.6	0.6 ± 1.6	0.30
Emergency CS	0.9 ± 1.3	1.1 ± 1.4	0.22	0.9 ± 1.2	1.2 ± 1.3	0.15	0.8 ± 1.8	0.7 ± 1.6	0.92
Elective CS	1.1 ± 1.5	1.1 ± 1.4	0.94	1.4 ± 1.4	1.1 ± 1.2	0.21	0.5 ± 1.5	0.8 ± 1.6	0.32
Preeclampsia	1.2 ± 0.5	1.1 ± 1.4	0.81	1.6 ± 0.1	1.2 ± 1.3	0.77	1.1 ± 0.6	0.8 ± 1.6	0.84
PIH	1.5 ± 1.2	1.0 ± 1.4	0.12	1.6 ± 1.2	1.2 ± 1.3	0.24	1.2 ± 1.3	0.8 ± 1.6	0.51
HDP	1.5 ± 1.1	1.0 ± 1.4	0.09	1.6 ± 1.1	1.2 ± 1.3	0.16	1.2 ± 1.0	0.7 ± 1.6	0.50
APH	1.5 ± 0.7	1.1 ± 1.4	0.53	1.0 ± 0.1	1.2 ± 1.3	0.86	2.1 ± 0.8	0.7 ± 1.6	0.21
Polyhydramnios	0.8 ± 1.7	1.1 ± 1.4	0.50	0.8 ± 1.8	1.2 ± 1.3	0.44	0.6 ± 0.2	0.8 ± 1.6	0.80
Oligohydramnios	2.1 ± 0.4	1.1 ± 1.4	0.21	N/A	N/A	N/A	2.1 ± 0.4	0.7 ± 1.6	0.09
Composite maternal	1.0 ± 1.3	1.1 ± 1.4	0.68	1.0 ± 1.2	1.2 ± 1.3	0.28	0.9 ± 1.5	0.7 ± 1.6	0.55
*Infant*									
Prematurity	1.1 ± 0.5	1.1 ± 1.4	0.92	1.0 ± 0.5	1.2 ± 1.3	0.68	1.5 ± 0.3	0.7 ± 1.6	**0.02**
Macrosomia	0.9 ± 1.2	1.1 ± 1.4	0.22	0.7 ± 1.2	1.3 ± 1.3	** < 0.01**	1.3 ± 1.3	0.6 ± 1.6	0.06
LGA	1.1 ± 1.4	1.1 ± 1.4	0.91	1.0 ± 1.4	1.2 ± 1.3	0.54	1.3 ± 1.2	0.7 ± 1.6	0.37
SGA	1.4 ± 1.4	1.0 ± 1.4	0.09	1.4 ± 1.4	1.1 ± 1.3	0.21	1.4 ± 1.2	0.7 ± 1.6	0.19
NICU admission	1.3 ± 1.2	1.0 ± 1.4	0.30	1.2 ± 1.3	1.2 ± 1.3	0.77	1.3 ± 0.7	0.7 ± 1.6	**0.04**
Jaundice	1.3 ± 0.5	1.1 ± 1.4	0.61	1.3 ± 0.9	1.2 ± 1.3	0.91	1.3 ± 0.4	0.7 ± 1.6	**0.04**
Neonatal death	1.0 ± 0.8	1.1 ± 1.4	0.98	1.0 ± 0.8	1.2 ± 1.3	0.80	N/A	N/A	N/A
Shoulder dystocia	2.0 ± 0.1	1.1 ± 1.4	**0.04**	N/A	N/A	N/A	2.0 ± 0.1	0.7 ± 1.6	** < 0.01**
Composite neonatal	1.1 ± 1.3	1.0 ± 1.4	0.51	1.1 ± 1.3	1.2 ± 1.3	0.21	1.3 ± 1.1	0.4 ± 1.7	** < 0.01**

SD: standard deviation; Yes: outcome present in this cohort; No: outcome not present in this cohort; APH: antepartum haemorrhage; CS: caesarean section; GDM: gestational diabetes; HDP: hypertensive disorders of pregnancy; LGA: large for gestational age; NGT: normal glucose tolerance; NICU: neonatal intensive care unit; PIH: pregnancy-induced hypertension; PPH: postpartum haemorrhage; SGA: small for gestational age; N/A: insufficient cases for statistical analysis

Bold values denote statistical significance at the *p* < 0.5 level

In the total cohort, T1 pGCD59 levels were higher in women whose infants experienced shoulder dystocia at birth (4.7 ± 0.03 vs. 3.6 ± 1.1 SPU, p < 0.01) compared to those that did not develop this outcome; T2 pGCD59 was significantly higher in women that developed polyhydramnios compared to those that did not ( 2.9 ± 1.1 vs. 2.5 ± 1.1 SPU, *p* = 0.03) and significantly lower in women that developed oligohydramnios (1.7 ± 0.03 vs. 2.5 ± 1.1 SPU, *P* < 0.01) and in women whose infants required NICU admission (2.3 ± 0.8 vs.2.6 ± 1.1 SPU, *p* = 0.04).

In the NGT cohort, T1 pGCD59 was significantly lower in women whose infants were macrosomic (3.3 ± 0.9 vs. 3.7 ± 1.1 SPU, *p* = 0.01) or LGA (3.2 ± 1.1 vs.3.7 ± 1.1 SPU, *p* = 0.04) and T2 pGCD59 was significantly higher in women that developed polyhydramnios (2.8 ± 0.4 vs. 2.4 ± 1.0 SPU, *p* = 0.04) compared to those that did not.

In the GDM cohort, T1 pGCD59 was significantly higher in women whose infants were macrosomic (4.2 ± 0.7 vs. 3.5 ± 1.0 SPU, *p* < 0.01) or LGA (4.3 ± 0.3 vs. 3.6 ± 1.0 SPU, *p* = 0.01). Levels of T2 pGCD59 were significantly higher in women that developed polyhydramnios (3.5 ± 0.1 vs. 2.8 ± 1.3 SPU, *p* < 0.01) and significantly lower in women who developed oligohydramnios (17 ± 0.03 vs. 2.8 ± 1.3 SPU, *p* < 0.01), APH (0.9 ± 0.02 vs. 2.9 ± 1.3 SPU, *p* < 0.01), and in women whose infants required NICU admission (2.2 ± 0.7 vs. 2.9 ± 1.3, *p* = 0.03) or jaundice (2.5 ± 0.1 vs. 2.8 ± 1.3, *p* = 0.04).

We also analysed the relationship between the changes between trimester 1 and trimester 2 in pGCD59 levels (*Δ*pGCD59) and adverse pregnancy outcomes. In the total cohort, ΔpGCD59 levels were higher in women whose infants experienced shoulder dystocia at birth (2.0 ± 0.1 vs. 1.1 ± 1.4 SPU, *p* = 0.04). In the NGT cohort, *Δ*pGCD59 were significantly lower in women who delivered a macrosomic baby (0.7 ± 1.2 vs. 1.3 ± 1.3 SPU, p < 0.01). In the GDM cohort, ΔpGCD59 was higher in women’s whose infants were born prematurely (1.5 ± 0.3 vs. 0.7 ± 1.6 SPU, *p* = 0.02), were admitted to NICU (1.3 ± 0.7 vs. 0.7 ± 1.6 SPU, p = 0.04) or had postpartum jaundice (1.3 ± 0.4 vs. 0.7 ± 1.6, *p* = 00.04)).

We further assessed the ability of T1 pGCD59 and T2 pGCD59 to predict the development of adverse pregnancy outcomes (Table 4). T1 pGCD59 generated very good AUCs for preeclampsia (AUC: 0.95, 95%CI: 0.90–0.99), oligohydramnios (AUC: 0.96, 95%CI: 0.93–0.98), neonatal death (AUC: 0.91, 95%CI: 0.78–0.99), and shoulder dystocia (AUC: 0.99, 95%CI: 0.98–0.99); similar results were found for ΔpGCD59. T2 pGCD59 generated very good AUCs for preeclampsia (AUC: 0.86–0.99) and oligohydramnios (AUC: 0.95, 95%CI 0.92–0.99). Optimal cut-off values for T1 and T2 pGCD59 (Youden’s index) with diagnostic accuracy measures for maternal and infant outcomes are presented in Table 5.

**Table 4:** pGCD59—adjusted ROC curves for GDM status, maternal age, BMI, maternal ethnicity, parity, previous GDM, and family history of diabetes at Galway University Hospital, Galway, Ireland, between November 2018 and March 2020, *n* = 378

Outcome	aROC	95% CI	*p*-value
T1 pGCD59			
*Mother*			
PPH	0.62	0.55–0.68	< 0.001
Preeclampsia	0.95	0.90–0.99	0.007
PIH	0.85	0.78–0.91	< 0.001
HDP	0.81	0.73–0.89	< 0.001
APH	0.77	0.66–0.87	0.032
Polyhydramnios	0.73	0.59–0.86	0.009
Oligohydramnios	0.96	0.93–0.98	0.024
Composite maternal	0.69	0.62–0.75	< 0.001
*Infant*			
Prematurity	0.68	0.55–0.82	0.021
Macrosomia	0.66	0.58–0.74	< 0.001
LGA	0.71	0.62–0.81	< 0.001
SGA	0.65	0.57–0.74	< 0.001
NICU admission	0.64	0.56–0.73	0.002
Jaundice	0.86	0.78–0.93	0.002
Neonatal death	0.91	0.78–0.99	0.043
Shoulder dystocia	0.99	0.98–0.99	0.017
Composite neonatal	0.60	0.54–0.66	0.001
T2 pGCD59			
*Mother*			
PPH	0.63	0.56–0.69	< 0.001
Preeclampsia	0.93	0.86–0.99	0.009
PIH	0.80	0.73–0.88	< 0.001
HDP	0.77	0.69–0.85	< 0.001
APH	0.83	0.72–0.93	0.006
Polyhydramnios	0.69	0.53–0.85	0.038
Oligohydramnios	0.95	0.92–0.99	0.026
Composite maternal	0.68	0.62–0.74	< 0.001
*Infant*			
Prematurity	0.61	0.41–0.80	0.028
Macrosomia	0.63	0.56–0.71	0.001
LGA	0.66	0.57–0.75	0.002
SGA	0.61	0.52–0.71	0.017
NICU admission	0.60	0.51–0.68	0.040
Jaundice	0.67	0.45–0.89	0.017
Neonatal death	0.88	0.72–0.99	0.063
Shoulder dystocia	0.77	0.68–0.85	0.010
Composite neonatal	0.59	0.53–0.65	0.003
ΔpGCD59			
*Mother*			
PPH	0.63	0.56–0.69	< 0.001
Preeclampsia	0.96	0.92–0.99	0.006
PIH	0.81	0.73–0.89	< 0.001
HDP	0.77	0.69–0.86	< 0.001
APH	0.76	0.59–0.92	0.046
Polyhydramnios	0.71	0.53–0.88	0.017
Oligohydramnios	0.98	0.98–0.99	0.010
Composite maternal	0.69	0.62–0.75	< 0.001
*Infant*			
Prematurity	0.62	0.44–0.81	0.012
Macrosomia	0.65	0.58–0.73	< 0.001
LGA	0.70	0.60–0.80	< 0.001
SGA	0.64	0.55–0.73	0.003
NICU admission	0.59	0.50–0.68	0.041
Jaundice	0.77	0.56–0.98	0.021
Neonatal death	0.91	0.81–0.99	0.043
Shoulder dystocia	0.97	0.96–0.99	0.027
Composite neonatal	0.60	0.53–0.65	0.003

aROC: adjusted receiver operator curve; APH: antepartum haemorrhage; CI: confidence interval; CS: caesarean section; GDM: gestational diabetes; HDP: hypertensive disorders of pregnancy; LGA: large for gestational age; NICU: neonatal intensive care unit; PIH: pregnancy-induced hypertension; PPH: postpartum haemorrhage; SGA: small for gestational age

**Table 5:** T1 pGCD59 and T2 pGCD59 optimal cut-off values (Youden’s index) and diagnostic accuracy measures

Outcome	pGCD59 cut-off level (SPU)	Sensitivity% (95%CI)	Specificity% (95%CI)	PPV (95%CI)	NPV (95%CI)
**T1 pGCD59**					
**Mother**					
PPH	3.3	47.0 (36.9–57.2)	61.0 (54.8–67.0)	31.8 (26.4–37.3)	74.9 (70.8–78.6)
Preeclampsia	5.5	33 (0.8–90.6)	96.9 (94.7–98.5)	8.3 (1.6–33.3)	99.4 (98.8–99.7)
PIH	4.3	85.0 (62.1–96.8)	30.4 (25.7–35.6)	6.6 (5.5–7.9)	97.2 (92.5–99.0)
HDP	4.3	82.6 (61.2–95.0)	30.4 (25.6–35.6)	7.3 (6.1–8.8)	96.3 (91.4–98.5)
APH	4.0	100.0 (47.8–100)	39.9 (34.9–45.2)	2.2 (2.1–2.4)	100.0
Polyhydramnios	2.4	72.7 (39.0–94.0)	11.7 (8.6–15.6)	2.8 (2.1–3.6)	95.5 (85.3–98.7)
Oligohydramnios	3.5	100.0 (15.8–100)	46.4 (41.2–51.7)	1.0 (0.9–1.1)	100.0
**Infant**					
Prematurity	3.8	75.0 (42.8–94.5)	46.3 (41.0–51.7)	4.5 (3.3–6.3)	98.2 (95.3–99.3)
Macrosomia	3.8	64.5 (51.3–76.3)	46.0 (40.3–51.8)	19.8 (16.6–23.4)	86.2 (81.4–90.0)
LGA	3.5	59.3 (40.6–76.3)	56.9 (51.4–62.4)	11.8 (8.9–15.4)	93.5 (90.4–95.7)
SGA	4.2	42.5 (27.0–59.1)	68.6 (63.3–73.7)	14.4 (10.2–20.0)	90.6 (87.9–92.7)
Jaundice	3.4	100.0 (54.1–100.0)	43.8 (38.3–48.9)	2.9 (2.7–3.2)	100.0
Shoulder dystocia	4.7	100.0 (15.8–100)	83.3 (79.1–87.0)	3.2 (2.6–4.0)	100.0
**T2 pGCD59**					
**Mother**					
PPH	2.6	66.6 (56.5–75.8)	46.0 (39.9–52.2)	31.7 (28.0–35.7)	78.3 (72.8–83.2)
Preeclampsia	3.0	66.6 (9.4–99.2)	75.0 (70.3–79.4)	2.1 (0.9–4.7)	99.6 (98.2–99.9)
PIH	2.6	89.4 (66.9–98.7)	41.9 (36.7–47.3)	7.7 (6.5–9.0)	98.7 (95.2–99.6)
HDP	2.6	81.8 (59.7–94.8)	41.7 (36.5–47.1)	7.7 (6.1–9.6)	96.7 (93.0–98.5)
APH	0.9	50.0 (11.8–88.2)	96.4 (94.0–98.1)	15.4 (4.8–39.4)	98.9 (98.1–99.4)
Polyhydramnios	2.5	90.9 (58.7–99.8)	51.2 (46.0–56.5)	5.4 (4.4–6.6)	99.5 (96.6–99.9)
Oligohydramnios	1.7	100.0 (15.8–100)	79.7 (75.3–83.7)	2.6 (2.1–3.2)	100.0
**Infant**					
Prematurity	2.8	91.6 (61.5–99.8)	32.8 (28.0–38.0)	4.4 (3.7–5.2)	99.1 (94.5–99.9)
Macrosomia	3.0	35.4 (23.7–48.7)	76.2 (71.0–80.9)	23.4 (17.1–31.1)	85.2 (82.6–87.5)
LGA	2.5	72.7 (54.5–86.7)	49.1 (43.6–54.6)	12.4 (10.1–15.1)	94.8 (91.1–97.0)
SGA	3.1	87.5 (73.2–95.8)	23.7 (19.2–28.7)	12.4 (11.0–13.8)	93.9 (86.9–97.3)
Jaundice	2.2	100.0 (54.1–100)	39.2 (34.2–44.5)	2.7 (2.5–2.9)	100.0
Shoulder dystocia	2.6	100.0 (15.8–100)	57.0 (51.8–62.2)	1.3 (1.1–1.4)	100.0

*APH*: Antepartum haemorrhage; *CI*: confidence interval; *HDP*: hypertensive disorders of pregnancy; *LGA*: large for gestational age; *NPV*: negative predictive value; *PIH*: pregnancy-induced hypertension; *PPH*: postpartum haemorrhage; *PPV*: positive predictive value; *SGA*: small for gestational age

**Fig. 2:**
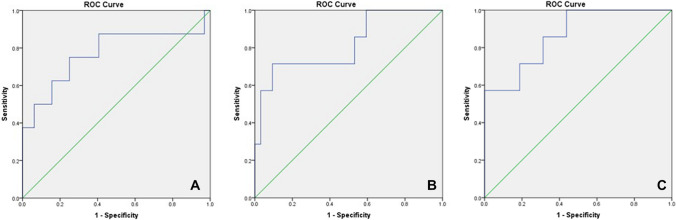
pGCD59—adjusted ROC curves for prematurity, small for gestational age and gestational diabetes in predicting neonatal hypoglycaemia at Galway University Hospital, Galway, Ireland, between November 2018 and March 2020, n = 41. A: T1 pGCD59 prediction of neonatal hypoglycaemia AUC: 0.77 95%CI: 0.54–0.99; B: T2 pGCD59 prediction of neonatal hypoglycaemia AUC: 0.81 95%CI: 0.62–0.99; C: ΔpGCD59 prediction of neonatal hypoglycaemia AUC: 0.86 95%CI: 0.72–0.99

In our cohort, we had post-partum glycaemic status on 41 infants (10.8%), 9 of whom had NH (21.9%). Of the infants that developed NH, 33.3% were born of mothers with GDM (*n* = 3). We calculated ROC curves adjusted for prematurity, SGA and GDM status. T1 pGCD59 predicted NH with an AUC of 0.77 (95%CI: 0.54–0.99); T2 pGCD59 predicted NH with an AUC of 0.81 (95%CI: 0.62–0.99); and ΔpGCD59 predicted NH with and AUC of 0.86 (95%CI: 0.72–0.99) (Fig. 2).

## Discussion

This is the first study exploring the relationship between T1 pGCD59, T2 pGCD59 and ΔpGCD59 levels and maternal and infant adverse pregnancy outcomes. pGCD59 has been investigated as a glycaemic marker for hyperglycaemia in pregnancy showing promising results. Ghosh et al*.* [19] found that T2 pGCD59 could identify GDM cases with an AUC of 0.92 (95%CI: 0.87–0.96) in a pregnant population screened for GDM with the 2-step process (glucose challenge test (GCT) followed by a 3 h 100 g OGTT diagnosed employing the Carpenter and Coustan criteria [35]). As pGCD59 is an emerging biomarker for the detection of hyperglycaemia in pregnancy, trimester-specific reference intervals for pGCD59 have not yet been established but will be investigated in future studies. However, given the high accuracy of pGCD59 to recognize GDM, we hypothesized that pGCD59 might potentially identify adverse pregnancy outcomes, in particular, GDM-related adverse pregnancy outcomes.

Ghosh et al*.* [19] found that higher maternal pGCD59 levels were associated with a higher prevalence of LGA. Interestingly, only a fifth of LGA cases in their study were born of women with GDM, while the remaining LGA cases were born of women who failed the GCT but did not meet the criteria for GDM diagnosis. A possible explanation for this is the treatment-effect, women with a formal GDM diagnosis receiving treatment thus reducing the risk of adverse outcomes, a finding supported by other studies [36, 37]. Furthermore, the women who failed the GCT but did not meet the diagnostic criteria for GDM in the Ghosh study might meet the threshold for GDM should the 2 h 75 g OGTT and 2013 WHO criteria be employed. The rationale being that the population identified using this approach would include milder cases of GDM consequent to the one-step protocol and lower diagnostic thresholds. This probably explains the discrepancy in our results as we did not find a significant difference in T2 pGCD59 between women who delivered an LGA infant and those who did not. We did, however, find higher levels T1 pGCD59 in women with GDM that delivered an LGA infant compared to women with GDM that did not have an LGA infant. A possible explanation for higher T1 pGCD59 levels compared with the T2 levels is the lifestyle improvements (diet, exercise, smoking, alcohol cessation, etc.) some women implement once they find out they are pregnant. This is also reflected in the higher ΔpGCD59 in women with GDM that delivered an LGA infant showing a higher reduction in pGCD59 levels between the first and second trimester.

Ma et al*.* [20] also explored the relationship between GDM status, LGA cases and pGCD59 sampled at < 20 WG in a pregnant population with a BMI ≥ 29 kg/m^2^. The team found that early pGCD59 (< 20 WG) can identify early GDM with an AUC of 0.86 (95%CI: 0.83–0.90). The mean pGCD59 levels in women with GDM that delivered an LGA infant were 4.3 ± 1.2 SPU, in NGT women that delivered an LGA infant was 2.7 ± 0.9 SPU, and in the total cohort of women that delivered an LGA infant were 3.2 ± 1.2 SPU similar to our T1 pGCD59 levels for the same groups (GDM 4.3 ± 0.3 SPU; NGT 3.2 ± 0.9 SPU, total cohort 3.4 ± 1.1 SPU).

We not only found higher levels of T1 pGCD59 in women with GDM that delivered an LGA infant, but also in women with GDM that delivered a macrosomic infant or an infant who suffered shoulder dystocia. These findings are plausible and complimentary considering the pathophysiology link between LGA, macrosomia, and shoulder dystocia in the context of GDM. Given the predictive ability of early pGCD59 to identify GDM described by Ma et al*.,* it is conceivable that early pGCD59 will be elevated in women with a baseline glycaemic/metabolic profile that predisposes to specific pregnancy complications.

In contrast with the T1 pGCD59 results, T2 pGCD59 was only elevated in women that developed polyhydramnios reflecting perhaps a degree of hyperglycaemia elevated enough to lead to an osmotic shift and accumulation of fluid but not sufficiently elevated to impact foetal development. In our GDM cohort, T2 pGCD59 levels were significantly lower in women who experienced APH, who developed oligohydramnios, or who delivered an infant that developed jaundice or required NICU admission. This seems counterintuitive as one would expect higher levels of glycation in an acute stress state. However, the pGCD59 was collected at 24–28 WG while the infant outcomes developed weeks later, and unfortunately, we had no data on what WG the APH or oligohydramnios occurred. Given the very low levels of T2 pGCD59 in women with GDM that experienced APH and the very small cases in our cohort, it is plausible that these results are the consequence of placental pathology leading to APH/oligohydramnios and NICU admissions and the maternal fluid replacements may have interfered with the levels of T2 pGCD59. Jaundice could be explained by maternal iron supplementation, especially in the context of haemorrhage [38].

PGCD59 has been studied in relation to adverse pregnancy outcomes in a cohort of women with T1DM by Meek et al*.* [21]. T1 pGCD59 generated the following AUCs for pregnancy outcomes: preeclampsia 0.56; prematurity 0.56; LGA 0.56; NH 0.61; and NICU admissions 0.56. T2 pGCD59 generated the following AUCs for pregnancy outcomes: preeclampsia 0.68; prematurity 0.64; LGA 0.59; NH 0.72; and NICU admission 0.73. While a direct comparison between the AUCs generated in our study and the study by Meek et al*.* is not suitable due to the difference in the populations studied (general population vs. women with T1DM), there are similar AUCs values generated for several outcomes. The biggest discrepancy resides in the prediction of preeclampsia, our study finding a very good predictive capacity of T1 and T2 pGCD59 in identifying preeclampsia cases. The most likely explanation is the very few cases of preeclampsia in our study leading to the statistical generation of high AUC values.

T1 pGCD59 generated a better AUC for the detection of LGA compared with T2 pGCD59 complimenting the findings in our pGCD59 levels distribution analysis. However, the overall predictive ability of pGCD59 to predict LGA/SGA/macrosomia/NICU admissions was poor to fair. T2 and Δ pGCD59 predicted NH with good accuracy. Hyperglycaemia in pregnancy leads to a transplacental transfer of glucose to the foetus which leads to foetal hyperglycaemia and hyperinsulinemia. This, in turn, causes neonatal hypoglycaemia at birth due to the loss of glucose from the mother and remaining elevated insulin levels. While our analysis was adjusted for GDM status, this does not exclude a degree of glycaemia below the current diagnosis thresholds detected by pGCD59 that still impacts on the infant pancreatic function. Our study also found a good prediction ability of T1 and T2 pGCD59 to identify preeclampsia/PIH/HDP. The relationship between GDM and hypertensive disorders is well documented [39, 40]. pGCD59 appears to have good predictive ability to identify women with a high-risk metabolic profile.

Where the case numbers allowed for statistical analysis, we also presented the optimal cut-offs for T1 and T2 pGCD59 together with diagnostic accuracy measures for predicting adverse pregnancy outcomes. While different cut-off levels displayed variable sensitivity and specificity depending on the outcome investigated, the NPV for both T1 and T2 pGCD59 for all outcomes was consistently high. This suggests a possible role for pGCD59 to identify women at low risk to develop pregnancy complications. Studies with a larger number of cases are required to confirm our findings.

The study has several limitations. The number of cases was very small for certain pregnancy outcomes, and the results of our analysis must be interpreted with caution. We did not include GDM as a pregnancy outcome as the data on the relationship between pGCD59 and GDM status are part of a separate study. Our study lacked ethnical diversity limiting the generalizability of our findings. We did not have data on the third-trimester pGCD59 which might better reflect the pathophysiologic changes leading to adverse pregnancy outcomes. Finally, the COVID-19 pandemic had a significant impact on this study through the recurring lockdowns, closure and reopening of laboratories with limited staff allowed to work on a restricted schedule and delays in procurement of laboratory consumables. This has compelled us to review and deviate from the original study protocol resulting in a reduction in the number of participant’s samples analysed.

## Conclusion

Early identification of women at risk of developing adverse pregnancy outcomes would allow for close monitoring, early intervention, and installations of suitable mechanisms to ensure a safe birth. This would not only lead to a reduction in complications with increased health and well-being for both mother and child, but it would also lead to a reduction in healthcare costs by reducing hospitalizations, length of stay, and admissions to intensive care units. This study has shown that pGCD59 has the potential to identify the development of adverse pregnancy outcomes. However, due to the low number of cases for certain outcomes, a definitive conclusion cannot be drawn. The results to date show promise and additional prospective studies with a larger number of cases are necessary to fully explore and validate the potential of this emerging biomarker in predicting adverse pregnancy outcomes.

## Data Availability

The datasets used and/or analysed during the current study are available from the corresponding author on reasonable request.

## Abbreviations

APH: Antepartum haemorrhage
AUC: Area under the curve
BMI: Body mass index
CI: Confidence interval
CV: Coefficient of variability
DBP: Diastolic blood pressure
EDTA: Ethylenediaminetetraacetic acid
ELISA: Enzyme-linked immunosorbent assay
GDM: Gestational diabetes
HDP: Hypertensive disorders of pregnancy
IUGR: Intrauterine growth restriction
LGA: Large for gestational age
NGT: Normal glucose tolerance
NH: Neonatal hypoglycaemia
NICU: Neonatal intensive care unit
OGTT: Oral glucose tolerance test
PIH: Pregnancy-induced hypertension
PPH: Postpartum haemorrhage
ROC: Receiver operating characteristics
SBP: Systolic blood pressure
SGA: Small for gestational age
T1DM: Type 1 diabetes
TOP: Termination of pregnancy
WG: Weeks of gestation
WHO: World Health Organization
